# Characterization of NF-kB-mediated inhibition of catechol-*O*-methyltransferase

**DOI:** 10.1186/1744-8069-5-13

**Published:** 2009-03-16

**Authors:** Inna E Tchivileva, Andrea G Nackley, Li Qian, Sean Wentworth, Matthew Conrad, Luda B Diatchenko

**Affiliations:** 1 Center for Neurosensory Disorders, School of Dentistry, University of North Carolina, Chapel Hill, NC 27599-7455, USA; 2 Comprehensive Center for Inflammatory Disorders, School of Dentistry, University of North Carolina, Chapel Hill, NC 27599-7455, USA

## Abstract

**Background:**

Catechol-*O*-methyltransferase (COMT), an enzyme that metabolizes catecholamines, has recently been implicated in the modulation of pain. Specifically, low COMT activity is associated with heightened pain perception and development of musculoskeletal pain in humans as well as increased experimental pain sensitivity in rodents.

**Results:**

We report that the proinflammatory cytokine tumor necrosis factor α (TNFα) downregulates COMT mRNA and protein in astrocytes. Examination of the distal *COMT *promoter *(P2-COMT) *reveals a putative binding site for nuclear factor κB (NF-κB), the pivotal regulator of inflammation and the target of TNFα. Cell culture assays and functional deletion analyses of the cloned *P2-COMT *promoter demonstrate that TNFα inhibits *P2-COMT *activity in astrocytes by inducing NF-κB complex recruitment to the specific κB binding site.

**Conclusion:**

Collectively, our findings provide the first evidence for NF-κB-mediated inhibition of COMT expression in the central nervous system, suggesting that COMT contributes to the pathogenesis of inflammatory pain states.

## Background

Catechol-*O*-methyltransferase (COMT) metabolizes catecholamines and thereby acts as a key modulator of dopaminergic and adrenergic/noradrenergic neurotransmission [1,2]. Converging lines of evidence have revealed an important role of COMT in the etiology and pathogenesis of a wide variety of central nervous system (CNS) disorders [2-4]. Recently, COMT has also been implicated in the regulation of pain perception [5,6]. Myofacial pain patients exhibit lower COMT activity relative to controls [7], and COMT inhibition increases pain sensitivity in rodents by promoting catecholamine stimulation of β_2_- and β_3_-adrenergic receptors [8].

The COMT protein exists in two major forms: a shorter soluble form (S-COMT) and a longer membrane-bound form (MB-COMT). They are encoded from one gene by two mRNA transcripts (1.3 and 1.5 kb in human, 1.6 and 1.9 kb in rats) regulated by the proximal *P1 *and distal *P2 *promoters, respectively [9-11]. Only the longer transcript was found in the brain [12] with the predominant protein being MB-COMT. However, S-COMT protein is also expressed in the brain from the longer MB-COMT mRNA isoform *via *a leaky scanning mechanism [13]. Though their sequences are largely homologous, MB-COMT has approximately a 10-fold greater affinity for dopamine and noradrenaline relative to S-COMT [14]. Seven novel COMT mRNA variants have also been detected in brain, however, they likely to exist at much lower levels than the primary transcript [15]. Although recent reports describe a neuronal expression of COMT [16], it is primarily considered a glial enzyme [17-19].

A significant role for glia in mediating pain has been implicated by studies of patients with persistent pain conditions and animal models of pain [20-22]. Proinflammatory cytokines are produced and released by activated microglia and astrocytes in the CNS as well as by immune cells at the site of injury or inflammation. [23-26]. TNFα is widely considered to be the prototypic proinflammatory cytokine due to its principal role in initiating the cascade of cytokines and growth factors involved in the inflammatory response [20]. Tissue levels of TNFα have been correlated with pain report in a number of painful diseases [27-29]. TNFα activates NF-κB, which is the pivotal regulator of cellular inflammatory responses [30-32]. Specifically, the NF-κB pathway plays one of the major roles in injury or inflammation-evoked activation of astrocytes [23,33,34]. Within the nervous system, NF-κB is most frequently composed of two DNA-binding subunits (p65/Rel A and p50) that form a complex with the inhibitory subunit IκB which normally retains NF-κB within the cytoplasm of unstimulated cells [35]. Signal-induced phosphorylation, ubiquitination, and degradation of IκB triggers NF-κB nuclear translocation and DNA binding. Phosphorylation of IκB is mediated by the IκB kinase (IKK) complex, which consists of two catalytic subunits, IKKα and IKKβ, and the regulatory subunit IKKγ [36]. Gene knock-out studies have established an essential role for IKKβ in TNFα-induced activation of NF-κB [37].

A growing number of reports reveal a crucial role of NF-κB in nociception. NF-κB activity is increased in animal models of neuropathic and inflammatory pain [38-42]. A specific IKK inhibitor reverses heightened pain sensitivity to noxious (hyperalgesia) and normally innocuous stimuli (allodynia) [43]. Increased neuropathic and inflammatory pain is suppressed by pretreatment with an NF-κB inhibitor [39,44]. Interestingly, selective inactivation of NF-κB in glial cells or astrocytes leads to decreased pain and better functional recovery [45-47].

Despite increasing evidence for an important role of NF-κB in pain regulation, very few studies have addressed the mechanisms whereby this pathway exerts its effects on nociception [38,39,41,43,48]. We hypothesized that NF-κB regulates expression of COMT, an enzyme known to contribute to enhanced pain states. Thus, the present study explored the relationship between the NF-κB pathway and COMT expression in order to gain an understanding of the cellular mechanisms underlying inflammatory pain.

## Results

### TNFα inhibits endogenous COMT expression in astrocytes

**Figure 1:**
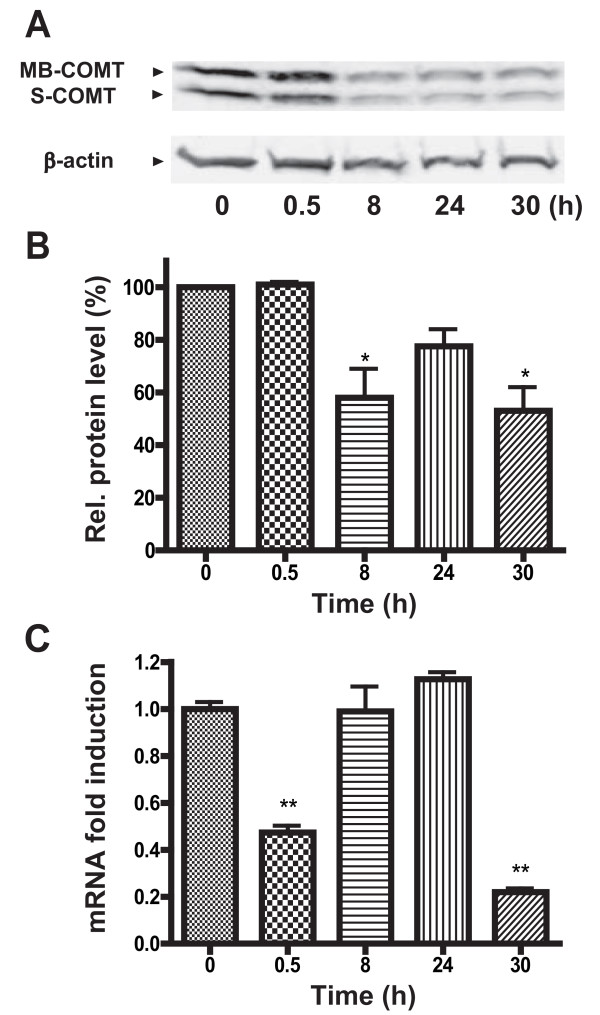
**COMT expression is downregulated by TNFα in primary astrocytes**. Administration of TNFα (20 ng/ml) decreases COMT protein expression in primary astrocytes as indicated by (A) a representative Western blot and (B) quantitative analysis of Western blots from experiments performed in triplicate. (C) Administration of TNFα (20 ng/ml) also decreases MB-COMT mRNA expression in primary astrocytes. Data are Means ± SEM. *P < 0.05 and **P < 0.01 different from untreated control.

To elucidate a potential role of TNFα in regulating COMT expression, we treated rat primary astrocytes with TNFα and measured COMT protein levels using Western blot analysis. A significant reduction in COMT protein expression was observed beginning at 8 h (Figure 1A) with a 60% maximal decrease relative to untreated control (*P *< 0.05; Figure 1B). Using quantitative real-time RT-PCR analysis, we further demonstrated a down-regulation of MB-COMT mRNA at 0.5 h and 30 h following TNFα treatment (*P *< 0.01; Figure 1C). This oscillatory rather than linear pattern of MB-COMT mRNA expression is in agreement with previously reported characteristics of NF-kB-mediated gene regulation and has been associated with autoregulation of NF-κB activity, as one of the genes activated by NF-κB is that encoding its own inhibitor, IκBα [49]. Finally, a dye exclusion test verified that the TNFα-dependent down-regulation of COMT was not due to cytotoxic effects as more than 95% of cells treated with TNFα remained viable (data not shown).

### Cloning and structural analysis of the human distal *COMT *promoter

**Figure 2:**
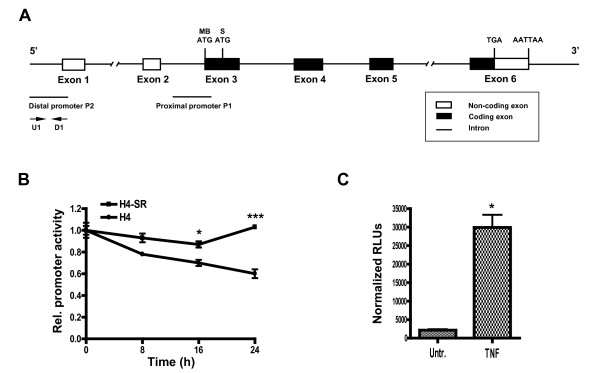
**Activity of *P2-COMT *promoter is downregulated by TNFα in H4 astroglial cells**. (A) Schematic diagram of human *COMT *gene. The exon-intron organization is not to scale. The positions of translation start codons for MB-COMT (MB-ATG) and S-COMT (S-ATG) polypeptides, translation stop codon (TGA), putative polyadenylation signal (AATTAA), promoter regions, and primers (U1 and D1) are shown. (B) Administration of TNFα (20 ng/ml) inhibits activity of transfected *P2-COMT/Luc *reporter in H4 but not in H4 IκBα-SR cells. Data are Means ± SEM. *P < 0.05, ***P < 0.001 H4 different from H4 IκBα-SR cells at 16 and 24 h, respectively. (C) TNFα-mediated inhibition is specific to *P2-COMT *promoter. The activity of *3x-κB/Luc *reporter (κB consensus sites from the MHC class I promoter) was tested after treatment with TNFα (20 ng/ml) for 24 h. *P < 0.05 different from untreated control.

To study the signaling mechanisms whereby TNFα regulates COMT expression, we cloned the human distal *COMT *promoter *(P2-COMT) *which controls transcription of MB-COMT mRNA (Figure 2A). A 1.5 kb DNA fragment corresponding to the previously published *P2-COMT *sequences [GenBank: Z26490 and AF001102] [11,50] was cloned in the pGL3 luciferase reporter vector. Using TFSearch database [51], we analyzed the cloned sequence and identified a number of potential transcription factor binding elements that can affect both constitutive and tissue-specific expression of COMT gene, such as TATA and CAAT boxes, CRE, C/EBP, and NF-κB sites. The presence of the NF-κB consensus binding site 5'-GGGGACGCCC-3' at position -109 from the first transcription initiation site of MB-COMT indicated that this promoter could be regulated by NF-κB pathway and respond to TNFα treatment.

### COMT inhibition by TNFα requires NF-κB activation

Luciferase reporter gene assays were employed to test the effect of TNFα treatment on *P2-COMT *activity. A chimeric human *P2-COMT/Luc *construct was transiently transfected into human H4 astroglioma cells. Consistent with the observed down-regulation of endogenous COMT expression, TNFα treatment decreased *P2-COMT *activity in time-dependent manner. After a 24-hour incubation with TNFα, *P2-COMT *activity was reduced to 60% relative to untreated control (*P *< 0.05 and *P *< 0.001 at 16 h and 24 h, respectively; Figure 2B). To test if the TNFα-mediated inhibition of *P2-COMT *activity requires NF-κB pathway activation, H4 astroglial cells stably transfected with IκBα super-repressor (SR), a nondegradable dominant-negative inhibitor of all NF-κB complexes, were transiently transfected with *P2-COMT/Luc *construct and *P2-COMT *activity was measured during 24 h of TNFα treatment. H4 IκBα-SR cells were no longer sensitive to TNFα-mediated inhibition of *P2-COMT *activity (Figure 2B).

As NF-κB is generally recognized as a positive regulator of gene expression, we used a reporter vector with a promoter known to be up-regulated in response to NF-κB activation. A luciferase reporter vector containing κB consensus sites from the MHC class I promoter was transfected into H4 astroglioma cells. After treatment with TNFα, the MHC promoter reporter showed a 14-fold increase in expression, demonstrating that the TNFα-mediated inhibition of COMT expression in astroglioma cells is *P2-COMT *promoter specific (*P *< 0.05; Figure 2C).

**Figure 3:**
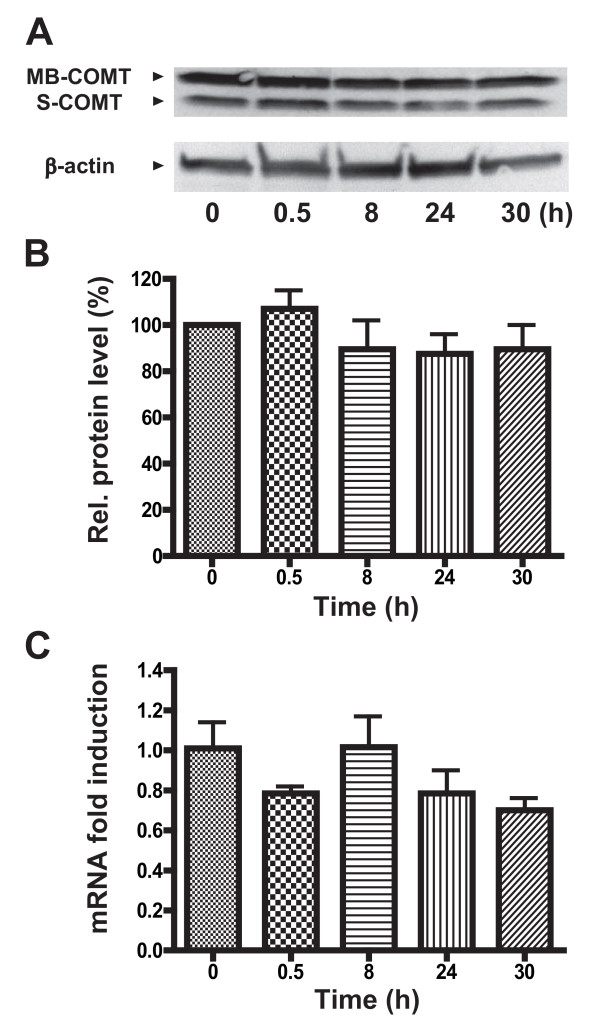
**TNFα-mediated repression of COMT is attenuated in H4 IκBα-SR astroglial cells**. Administration of TNFα (100 ng/ml) does not reduce COMT expression in H4 IκBα-SR cells as indicated by (A) a representative Western blot, (B) quantitative analysis of Western blots from experiments performed in triplicate, and (C) quantitative real-time RT-PCR analysis of MB-COMT mRNA. *P *> 0.05 different from untreated control.

Finally, to test effect of NF-κB pathway on endogenous COMT expression, we also treated H4 IκBα-SR cells with TNFα. Consistent with reporter assay results, TNFα was unable to significantly repress endogenous COMT protein and mRNA levels in H4 IκBα-SR cells (*P *> 0.05 for protein level and *P *> 0.05 for mRNA; Figure 3A, B, and 3C).

### TNFα inhibits COMT *via *a canonical NF-κB activation mechanism

**Figure 4:**
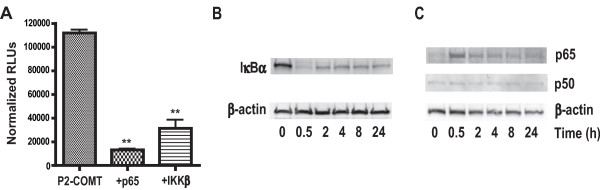
**NF-κB is required for COMT downregulation**. (A) Cotransfection of pCMV-SPORT-M-p65 or pCMV-SPORT-M-IKKβ inhibits *P2-COMT/Luc *reporter activity in H4 cells. Data are Means ± SEM. **P < 0.01 different from *P2-COMT/Luc *alone. Western blot analysis was performed with protein extracts from H4 cells treated with TNFα (20 ng/ml) for the indicated timepoints. Expression of IκBα was evaluated in cytoplasmic fraction of H4 cells (B), whereas p65 and p50 protein levels were tested in nuclear extracts (C).

As TNFα may function *via *NF-κB and JUN N-terminal kinase (JNK) signaling cascades [52], we sought to determine whether a signal that elicits NF-κB activation alone would repress *COMT *gene expression. Thus, we tested if overexpression of p65 or IKKβ, which are both essential for NF-κB activation, would negatively regulate *P2-COMT *activity. Our data demonstrate that p65 or IKKβ overexpression dramatically decreases *P2-COMT *activity (*P *< 0.01; Figure 4A).

Next, we examined whether TNFα engaged the canonical mechanism of IκBα phosphorylation and degradation to trigger transport of NF-κB to the nucleus in our experimental conditions. H4 cells were treated with TNFα and then cytoplasmic and nuclear extracts analyzed by Western blot. The degradation of IκBα in the cytoplasmic fraction of the cells and simultaneous increase in the nuclear level of p65 was observed at 30 min (Figure 4B and 4C), characteristic of dynamic NF-κB signaling [53]. Together, these results demonstrate that TNFα initiates the canonical IκBα degradation pathway to activate NF-κB in H4 astroglioma cells, and that NF-κB activation inhibits COMT in glial cells.

### Identification of the functional NF-κB binding site in the *P2-COMT *promoter

**Figure 5:**
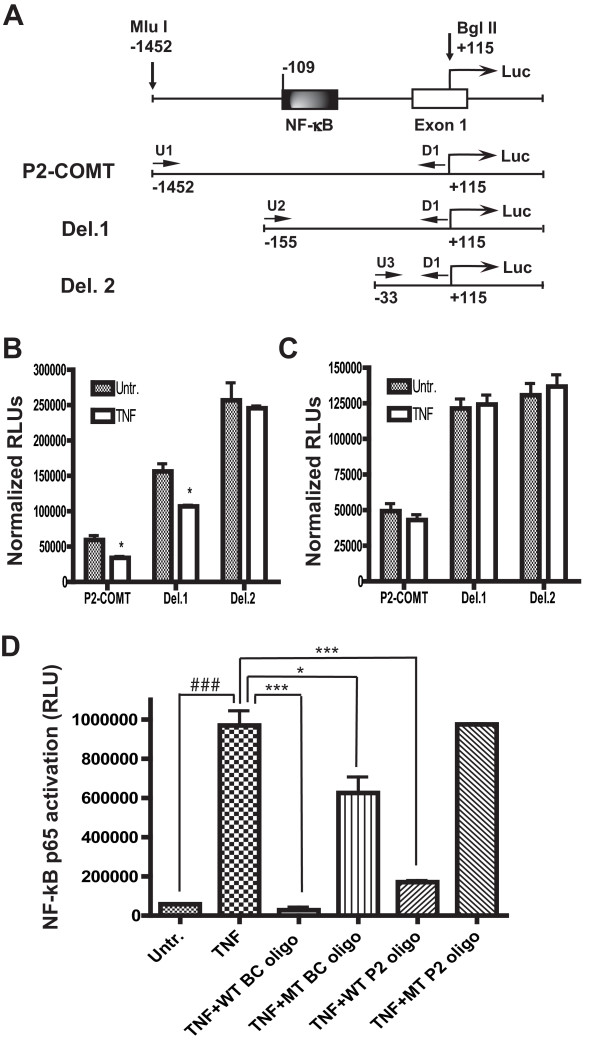
**Identification of TNFα-responsive region in the *P2-COMT *promoter**. (A) Schematic diagram of the *P2-COMT/Luc *construct and its serial deletions *Del.1 *and *Del.2*. The effect of TNFα (20 ng/ml, 8 h of treatment) on the relative activity of the *P2-COMT/Luc, Del.1 *and *Del.2 *constructs transfected into H4 (B) and H4 IκBα-SR (C) cells. Data are Means ± SEM. *P < 0.05 different from untreated control. (D) Binding of p65 to a plate-immobilized oligonucleotide containing a κB binding site was measured by TransAM NF-κB assay in the nuclear extracts from H4 cells 30 min post TNFα treatment. This activation was prevented by competition with a wild-type binding control (WT BC) κB consensus oligonucleotide or a wild-type *P2-COMT *κB consensus oligonucleotide (WT P2). Oligonucleotides with mutated κB binding sites – a mutant binding control (MT BC) and a mutant *P2-COMT *(MT P2-COMT) – had little or no effect. Data are Means ± SEM. ^###^*P *< 0.001 TNFα-treated different from untreated control, ***P < 0.001 TNFα-treated different from TNFα+WT BC and WT P2 oligos, *P < 0.05 TNFα-treated different from TNFα+MT BC oligo.

To identify the NF-κB-responsive region in the *P2-COMT *promoter, we analyzed the activity of serial 5'deletions of the *P2-COMT/Luc *constructs transiently transfected into H4 or H4 IκBα-SR cells (Fig. 5A). Consequent deletions of 5'fragments led to a graduate increase in overall basal promoter activity in all constructs, suggesting the presence of serial putative negatively regulating elements along the *P2-COMT *promoter. *Del.2 *construct that lack the putative κB consensus binding site also showed a lack of response to TNFα treatment in either H4 or H4 IκBα-SR cells (*P *> 0.05; Figure 5B and 5C). Conversely, TNFα treatment of H4 but not H4 IκBα-SR cells inhibited activity of construct *Del.1 *containing the putative κB consensus binding site and the initial *P2-COMT *promoter construct (*P *< 0.05 for H4 cells and *P *> 0.05 for H4 IκBα-SR cells; Figure 5B and 5C). Thus, the region between position -155 and -33 appears to be crucial for TNFα-dependent suppression of *P2-COMT *activity.

To determine whether TNFα induces NF-κB binding to the consensus DNA site of the identified region, we used ELISA to detect bound NF-κB p65 subunits. This method was employed as an alternative to the electrophoretic mobility shift assay, as it was reported to be more sensitive and quantitative [54]. DNA binding of p65 was dramatically increased in nuclear extracts of H4 cells after 30 min of TNFα treatment (*P *< 0.001; Figure 5D). This binding was abrogated by incubation of the nuclear extracts with either a competing control wild-type κB consensus oligonucleotide (Figure 5D, WT BC oligo) or with a competing oligonucleotide containing a wild-type putative κB binding sequence 5'-GGGGACGCCC-3' at position -109 in the *P2-COMT *promoter (Figure 5D, WT P2 oligo). Conversely, both oligonucleotides containing either a mutated version of the control κB consensus sequence (Figure 5D, MT BC oligo) or a mutated sequence 5'-GCTCACGCCC-3' of the putative κB binding site of the *P2-COMT *promoter (Figure 5D, MT P2 oligo) had a minimal effect on TNFα-induced p65 binding. Results from these experiments confirm that TNFα induces p65 recruitment to the functional κB binding site at position -109 in the *P2-COMT *promoter.

## Discussion

In the present report, we provide the first demonstration that *COMT *gene expression is downregulated by TNFα in primary rat astrocytes at both protein and mRNA levels. As the *P2-COMT *promoter controls expression of MB-COMT, the main COMT transcript in brain, this promoter was cloned from human genomic DNA and transfected in H4 astroglioma cells. The activity of the cloned promoter was substantially suppressed by TNFα in a time-dependent manner.

A number of putative regulatory elements have been described in *P1*- and *P2-COMT *promoters, including estrogen response (ER) elements [50] that likely mediate estradiol-dependent downregulation of COMT expression in cell culture [55]. *P2-COMT *also contains abundant methylation sites associated with cancer development [56], schizophrenia, and bipolar disorder [57]. We identified a novel putative regulatory site – a κB consensus sequence that is a potential target for TNFα-dependent NF-κB activation.

Next, we demonstrated that TNFα-dependent COMT downregulation was indeed mediated by the NF-κB pathway. Transient expression of p65, the essential component of NF-κB complexes, or IKKβ, the major positive regulator of NF-κB activition, significantly decreased *P2-COMT *reporter expression. In addition, H4 IκBα-SR cells lost the ability to regulate *P2-COMT *promoter expression in response to TNFα treatment. The TNFα-mediated suppression of endogenous COMT expression was also abrogated in H4 IκBα-SR cells. Moreover, we confirmed that TNFα activated NF-κB in H4 astroglioma cells through the canonical IκB degradation pathway to trigger p65 nuclear translocation and DNA binding.

Our data strongly suggest that the putative κB binding site 5'-GGGGACGCCC-3' at position -109 of the *P2-COMT *promoter region is a functional site for NF-κB-mediated regulation of COMT expression as deletion of the *P2-COMT *region containing this site abrogated TNFα-dependent inhibition of *P2-COMT *activity in H4 astroglioma cells. Furthermore, competition experiments performed with the wild type or mutant site-specific oligonucleotides showed that TNFα indeed induced recruitment of p65 to this κB consensus binding site of the promoter.

Although NF-κB-mediated activation of transcription is well known, the mechanisms of NF-κB-mediated repression are poorly established. Probably, the best studied example of transcriptional repression by NF-κB complex is described for Dorsal transcription factor, a *Drosophila *Rel family member that can either activate or repress gene expression through the recruitment of coactivators such as CBP or corepressors such as Groucho [58]. Furthermore, a number of examples have been reported in mammals. NF-κB can repress transcription by competing with steroid receptors for a common promoter *cis*-DNA element [59]*via *N-myc recruitment to the glutamate transporter gene promoter [53] and through inhibiting histone H4 acetylation at the cytochrome P-450 1A1 promoter [60]. Thus, further experiments should be conducted to address the specific mechanism underlying NF-κB-dependent inhibition of *COMT *gene expression. Interestingly, consequent deletions of 5'fragments of the *P2-COMT *promoter led to a significant increase in overall basal promoter activity. This result would suggest the presence of a number of putative negatively regulating elements along the *P2-COMT *promoter other than ER- and κB-response elements. Although, to date, no studies have systematically searched for regulators of COMT expression, this finding clearly warrants further research.

Our results demonstrating that COMT expression is downregulated in astrocytes under inflammatory conditions are in line with those of other studies showing a positive correlation between astrocyte activation and exaggerated pain responses [24,25,61]. Intrathecal injection of gp120 (human immunodeficiency virus-1 envelope glycoprotein) induces mechanical allodynia *via *the release of proinflammatory cytokines and NF-κB activation in spinal cord astrocytes, but not in microglial cells or neurons [62]. Selective inactivation of astroglial NF-κB in transgenic mice expressing a dominant negative form of the inhibitor IκBα leads to a dramatic improvement in functional recovery after contusive spinal cord injury (SCI) [46] and decreases formalin-induced pain [47]. Additionally, several recent studies report cell type-specific NF-κB activation by cytokines. For example, in rat brain cultures IL-1 induces NF-κB activation in astrocytes, but not in neurons [63,64]. Taken together, these studies unequivocally link NF-κB activation in astrocytes to pain states.

Although activation of the NF-κB pathway has been deemed critical for the development of pain [40-42], there are few reports studying NF-kB-dependent pro-nociceptive signaling. Historically, these studies have focused on NF-kB-dependent up-regulation of pro-inflammatory cytokines [25,65], cyclooxygenase-2 (COX-2) [39,43], inducible and neuronal nitric oxide synthases (iNOS and nNOS) [38,41], c-src [48], and c-fos [38]. However, recent studies from our group demonstrated that genetic variants of *COMT *coding for low enzymatic activity are associated with heightened experimental pain sensitivity and the onset of a myofacial pain condition in humans [5]. Additionally, pharmacologic inhibition of COMT in a rat model of inflammation resulted in elevated pain sensitivity [8]. Together, these data suggest that an NF-κB-mediated decrease in COMT expression is likely to contribute to heightened pain sensitivity under inflammatory conditions. A series of *in vivo *experiments further addressing this hypothesis are currently being conducted in our laboratory.

## Conclusion

Collectively, our results provide the first evidence that COMT expression is downregulated in astrocytes *via *recruitment of the NF-κB complex to a specific κB-site at the *P2-COMT *promoter. NF-κB-mediated inhibition of COMT in the CNS may represent a novel mechanism contributing to inflammatory pain. NF-κB is regarded as one of the most important targets for therapeutic intervention against inflammatory conditions [66,67]; thus, elucidating the cellular mechanisms that underlie NF-κB-mediated inflammatory pain will promote the development of novel therapies including pharmacologic agents that block COMT-dependent pain signaling.

## Methods

### Cell culture and reagents

Primary astrocytes were isolated and cultured as described earlier [68]. Human H4 astroglioma cells were obtained from ATCC (HTB-148) and cultured in DMEM (Sigma), 10% fetal bovine serum (FBS; HyClone) and 1× penicillin-streptomycin (Invitrogen). H4 cells stably expressing IκBα-SR were a generous gift from Dr. Baldwin (UNC) and generated as described previously [53]. All oligonucleotides were obtained from MWG-Biotech AG. The pCMV-SPORT-M, pCMV-SPORT-M-p65 and pCMV-SPORT-M-IKKβ expression vectors were a generous gift provided by Dr. Romanov (Attagene) and 3x-κB/luc construct was a gift from Dr. Baldwin (UNC).

### Quantitative real-time RT-PCR

Total RNA was isolated using the Trizol reagent (Invitrogen), treated with RNase free-DNase I (Promega) and reverse transcribed with random primers by Superscript III (Invitrogen). The cDNA was amplified with SYBR Green PCR master mix (Applied Biosystems) using forward and reverse PCR primers (5'-CCAGAGGAGACCCCAGACC-3' and 5'-ACAGCTGCCAACAGCAGAG-3', respectively, for human MB-COMT; 5'-GGAAATCGTGCGTGACATC-3' and 5'-CATGGATGCCAAGGATTC-3', for human β-actin; 5'-CCAGAGGAGACCCCAGACC and 5'-ACAGCTGCCAACAGCAGAG-3', for rat MB-COMT; and 5'-TGCGGGTCATAAGCTTGC-3' and 5'-CGATCCGAGGGCCTCACTA-3' for rat 18S rRNA) in S2 Real Time PCR machine (Eppendorf). PCR reactions were performed in triplicate. Three independent experiments were performed, and the result of a representative experiment is shown. MB-COMT mRNA levels were normalized to β-actin RNA or 18S rRNA as an endogenous control.

### Western blot analysis

10–50 μg of protein lysates from whole cells, nuclear and cytoplasmic extracts, normalized for protein content using a BCA Protein Assay Kit (Pierce), were run on precast Novex Tris-Glycine gels (Invitrogen), blotted onto nitrocellose (Whatman), and blocked in TBST with 5% nonfat dry milk. The following antibodies were used: COMT (Chemicon, AB5873), β-actin (I-19) (Santa Cruz, SC-1616), IκBα (C-21) (Santa Cruz, SC-371), p65 (Cell Signaling, #3034), and p50 (Santa Cruz, SC-7178). Chemiluminescence was detected in ImageQuant-ECL Imaging System (GE Healthcare) and images were analyzed using ImageQuant TL software (GE Healthcare). Blots from three independent experiments were densitometrically analysed and the values normalized to the β-actin control, with untreated group set to 100%.

### Cloning of human P2-COMT distal promoter

Primers **U1 **5'-CCTACGCGTGCTCCTCTGGCGGAAAGGAA-3' and **D1 **5'-CGAAGATCTACCTCTCCCGCGACGGCCCG-3', with added Mlu I and Bgl II restriction sites, respectively, were used to amplify *P2-COMT *from 50 ng of human genomic DNA with GeneAmp PCR kit (Applied Biosystems). The 1.5 kb PCR product was digested by Mlu I and Bgl II restrictases (NEB), gel-purified, ligated into pGL3 Luciferase Reporter Vector (Promega) using Rapid DNA Ligation Kit (Roche) and transformed into competent *E. coli *DH5α cells (Invitrogen). Recombinant plasmids were isolated using EndoFree Plasmid Kit (Qiagen) and sequenced at UNC sequencing facility. Putative regulatory elements were determined with TFSearch database .

### Construction of serial 5'-end deletions of human P2-COMT/Luc clone

Serial deletions were generated by PCR amplification of corresponding fragments from *P2-COMT/Luc *clone using forward primers, containing Mlu I restriction site, and reverse primers, containing Bgl II site, **U2 **5'-CCTACGCGTGCGGACACCCTCACGAGGACA-3' and **D1**, respectively, for *Del. 1*, and **U3 **5'-CCTACGCGTCCACCGGAAGCGCCCTCCTA-3' and **D1 **for *Del. 2*. The amplified fragments were digested by Mlu I and Bgl II, purified from the agarose gel and cloned into pGL3 reporter vector. Deletions were confirmed by sequencing. The nucleotide numeration was based on Tenhunen et al. [11].

### Transient transfection, luciferase and β-galactosidase assays

Cells were seeded into 12-well plates (5 × 10^4^cells/well) and transfected with 500 ng of total DNA using FuGene 6 reagent (Roche). Normally, up to 400 ng of P2-COMT luciferase reporter and 30 ng of control plasmid for transfection efficiency (pSV-β-galactosidase vector, Promega) were used for transfection. The amount of DNA was kept constant by addition of pCMV-Sport-M vector with no insert. Cells were treated by TNFα (R&D Systems) and harvested 48 h after transfection. Luciferase activity was determined using Luciferase Assay System (Promega) and normalized for transfection efficiency by measuring the β-galactosidase activity using a β-Galactosidase Enzyme Assay System (Promega). Transfections were performed in triplicate, and a representative experiment is shown.

### ELISA for activated NF-κB

NF-κB activation was measured using TransAM NF-κB p65 Chemi Kit (Active Motif). Cell lysates were tested for their ability to bind to a plate-immobilized oligonucleotide containing a κB consensus binding site (5'-GGGACTTTCC-3'). Competition experiments were performed with the wild-type (ACCGC**GGGGACGCCCGGGGACGCCC**CGACC) and mutant (5'-ACCGC**G**CTC**ACGCCCGCTCACGCCC**CGACC) oligonucleotides specific to *P2-COMT *κB binding site and κB wild-type and mutated consensus oligonucleotides provided by the manufacturer. The wild-type but not mutated oligonucleotides were expected to compete with NF-κB for binding. Chemiluminescence was measured in 1420 Multilabel Counter Victor3 (PerkinElmer). Nuclear extracts were prepared using Nuclear Extract Kit (Active Motif).

### Statistical Analysis

Protein, mRNA, and promoter activity data were analyzed by paired t-test and analysis of variance (ANOVA) with post-hoc tests. *P *< 0.05 was considered to be statistically significant.

## List of abbreviations

COMT: catechol-O-methyltransferase; IκBα: inhibitory factor κB; IKK: IκB kinase; NF-κB: nuclear factor κB; TNFα: tumor necrosis factor α.

## Competing interests

The authors declare that they have no competing interests.

## Authors' contributions

IET and LBD conceived of the study, and IET, AGN, SW, MC, and LQ performed experiments. IET, AGN, and LBD participated in writing of the manuscript. All authors read and approved the final manuscript.
